# The photon dose calculation algorithm used in breast radiotherapy has significant impact on the parameters of radiobiological models

**DOI:** 10.1120/jacmp.v15i4.4853

**Published:** 2014-07-08

**Authors:** Saskia Petillion, Ans Swinnen, Gilles Defraene, Karolien Verhoeven, Caroline Weltens, Frank Van den Heuvel

**Affiliations:** ^1^ Department of Radiation‐Oncology University Hospitals of Leuven KU Leuven Leuven Belgium; ^2^ Department of Radiation Oncology Maastricht University Medical Centre Maastricht The Netherlands; ^3^ Gray Institute for Radiation Oncology and Biology University of Oxford Oxford United Kingdom

**Keywords:** breast, algorithm, radiobiology, TCP, NTCP

## Abstract

The comparison of the pencil beam dose calculation algorithm with modified Batho heterogeneity correction (PBC‐MB) and the analytical anisotropic algorithm (AAA) and the mutual comparison of advanced dose calculation algorithms used in breast radiotherapy have focused on the differences between the physical dose distributions. Studies on the radiobiological impact of the algorithm (both on the tumor control and the moderate breast fibrosis prediction) are lacking. We, therefore, investigated the radiobiological impact of the dose calculation algorithm in whole breast radiotherapy. The clinical dose distributions of 30 breast cancer patients, calculated with PBC‐MB, were recalculated with fixed monitor units using more advanced algorithms: AAA and Acuros XB. For the latter, both dose reporting modes were used (i.e., dose‐to‐medium and dose‐to‐water). Next, the tumor control probability (TCP) and the normal tissue complication probability (NTCP) of each dose distribution were calculated with the Poisson model and with the relative seriality model, respectively. The endpoint for the NTCP calculation was moderate breast fibrosis five years post treatment. The differences were checked for significance with the paired t‐test. The more advanced algorithms predicted a significantly lower TCP and NTCP of moderate breast fibrosis then found during the corresponding clinical follow‐up study based on PBC calculations. The differences varied between 1% and 2.1% for the TCP and between 2.9% and 5.5% for the NTCP of moderate breast fibrosis. The significant differences were eliminated by determination of algorithm‐specific model parameters using least square fitting. Application of the new parameters on a second group of 30 breast cancer patients proved their appropriateness. In this study, we assessed the impact of the dose calculation algorithms used in whole breast radiotherapy on the parameters of the radiobiological models. The radiobiological impact was eliminated by determination of algorithm specific model parameters.

PACS numbers: 87.55.dh, 87.55.dk

## I. INTRODUCTION

The photon dose calculation algorithms in commercial treatment planning systems are classified in two groups, depending on the method used for inhomogeneity approximation: type‐a algorithms and type‐b algorithms.^(1)^ The inhomogeneity corrections used in the former are based on equivalent path length scaling — for example, the pencil beam convolution algorithm^(2)^ with modified Batho heterogeneity correction (PBC‐MB) or with equivalent tissue‐air‐ratio correction. Type‐b algorithms — for example, the collapsed‐cone convolution algorithm^(3)^ and the analytical anisotropic algorithm (AAA)^(4)^ — approximate also the lateral electron transport. Recently, a new algorithm, which solves the linear Boltzmann transport equation (LBTE), has been introduced: Acuros XB Advanced Dose Calculation algorithm (Varian Medical Systems, Inc., Palo Alto, CA).

Studies on the performance and accuracy of the dose calculation algorithms in inhomogeneous phantoms have revealed that the type‐b algorithms are superior to the type‐a algorithms,^5^, ^6^, ^7^, ^8^ and that the Acuros XB algorithm is an accurate alternative to Monte Carlo calculations.^(9)^ Type‐a and type‐b algorithms calculate absorbed dose to water. In contrast, Acuros XB calculates dose to medium, which can be converted to dose to water for treatment plan evaluation. However, the phantom study by Fogliata et al.^(9)^ has shown that the AAA percent‐depth‐dose (PDD) curves are close to the Acuros XB dose‐to‐medium PDD curves, but are significantly different from the Acuros XB dose‐to‐water PDD curves.

The impact of the algorithm on the target coverage in lung cancer treatment has been described.^1^, ^7^, ^8^, ^10^ Two recent studies have quantified the difference between AAA and Acuros XB in stereotactic body radiation therapy of non‐small cell lung cancer.^11^, ^12^ There is some knowledge on the dose distributions in target volumes close to air cavities like, for example, in the head and neck region.^1^, ^8^, ^13^ Recent planning studies discuss the impact of low‐density organs on the coverage of adjacent target volumes, as in breast cancer treatment.^7^, ^14^, ^15^, ^16^

More interesting than comparing the physical dose distributions is the investigation of the radiobiological (i.e., the clinical) impact of the observed differences. For lung cancer patients, the impact of the difference between the type‐a and the type‐b algorithms on the normal tissue complication probability (NTCP) of the lungs and the heart has been reported.^17^, ^18^ Also for breast cancer treatments, the impact has been studied, and algorithm specific NTCP parameters for the calculation of radiation pneumonitis have been derived.^(19)^ Recently, the radiobiological impact of Acuros XB compared to AAA in esophageal cancer treatment planning has been quantified for both the target volume and the organs at risk.^(20)^ Studies on the radiobiological impact of the dose calculation algorithm and of both dose‐reporting modes of Acuros XB on the prediction of the tumor control probability (TCP) and the NTCP of moderate breast fibrosis (${\text{NTCP}}_{\text{Fibrosis}}$) in whole breast radiotherapy (WBRT) are lacking.

This study therefore aims to: 1) assess the radiobiological impact (both on the TCP and on the ${\text{NTCP}}_{\text{Fibrosis}}$) of the algorithm used in WBRT; 2) eliminate the radiobiological impact by determination of algorithm specific TCP and ${\text{NTCP}}_{\text{Fibrosis}}$ parameters; and 3) validate the new parameters. For a large breast population, we compared a type‐a algorithm, a type‐b algorithm, and the Acuros XB algorithm, using dose‐to‐medium and dose‐to‐water dose reporting.

## II. MATERIALS AND METHODS

### A. Dose calculation algorithms

Three photon dose calculation algorithms commercially available in Eclipse (Varian Medical Systems, Inc.) were compared: PBC‐MB,^(2)^ AAA,^(4)^ and Acuros XB,^(21)^ all version 10.0.28. For the latter, both dose‐reporting modes were applied: dose to medium (Acuros‐M) and dose to water (Acuros‐W). The grid size was 0.25 cm for the PBC‐MB and AAA calculations, and 0.2 cm for the Acuros XB calculations.

In case of PBC‐MB and AAA dose calculation, the Hounsfield units (HU) of the CT scan are converted to electron density relative to water. The PBC‐MB algorithm is a convolution algorithm based on pencil beam kernels. First, the dose distribution in a homogeneous water‐equivalent medium is calculated. Next, the tissue inhomogeneities are taken into account by multiplying the dose distribution with correction factors. The inhomogeneity correction factor is calculated along the central axis with the modified Batho power law. AAA is an analytical dose calculation algorithm based on pencil beam convolution/superposition. Primary photons, scattered extra‐focal photons, and contaminating electrons and photons scattered from the beam limiting devices are separately modeled. In contrast to PBC‐MB, AAA models the lateral electron transport. Tissue heterogeneities are accounted for anisotropically in the full 3D neighborhood of an interaction site, according to the local electron density. Acuros XB explicitly solves the LBTE using numerical methods. The LBTE describes the macroscopic behavior of radiation particles in matter. In Eclipse, first the HUs of the CT scan are converted to mass density such that the patient's tissue properties can be taken into account. Next, the energy‐dependent angular electron fluence is calculated. Finally, the dose ($D_{i}$) in each grid voxel, i, is calculated based on the energy‐dependent angular electron fluence, the macroscopic electron energy deposition cross section, and the material density. If dose‐to‐medium dose reporting is selected, then $D_{i}$ is calculated using the material properties of voxel i. If dose to water is selected, then $D_{i}$ is calculated in a post‐processing step that considers the patient as consisting of water.

### B. Patients

Sixty patients, divided in two groups of 30 consecutive patients (15 left‐sided and 15 right‐sided), undergoing a breast treatment, were included in the study. For all patients, a free‐breathing CT scan in treatment position was reconstructed with 3 mm slice thickness (Somatom Sensation Open, Siemens Medical Solutions, Erlangen, Germany). Next, a physician delineated the breast tissue (${\text{CTV}}_{\text{Breast}}$) using the lead wire around the palpable breast tissue. A margin of 1 cm was used to create the planning target volume (${\text{PTV}}_{\text{Breast}}$).

### C. Treatment plans

**Figure 1:**
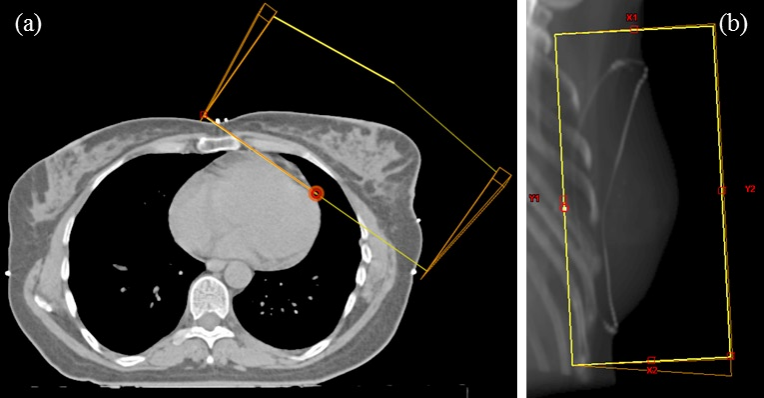
Single‐isocentric WBRT beam setup (a) and beam's eye view (b).

Single‐isocentric WBRT was performed with two wedged half‐beams to a total dose of 50 Gy in 2 Gy fractions (Fig. 1). Each clinical dose distribution, calculated with PBC‐MB, was recalculated using AAA, Acuros‐M and Acuros‐W with fixed monitor units (MUs). The fixed MU recalculation was repeated for PBC without heterogeneity correction, referred to as PBC‐Hom, for study purposes only (see Aim 2). The results of this study are based on the use of the same beam‐data set of a Varian Clinac 2100 C/D (Varian Medical Systems, Inc.).

### D. Aim 1: Assessment of algorithm impact on TCP and ${\text{NTCP}}_{\text{Fibrosis}}$

The differential dose‐volume histograms (dDVH) of the ${\text{CTV}}_{\text{Breast}}$ and the ${\text{PTV}}_{\text{Breast}}$ were exported to be imported in an in‐house developed MATLAB program (MathWorks, Natick, MA) for TCP and ${\text{NTCP}}_{\text{Fibrosis}}$ calculation, respectively.

Table 1 gives the main characteristics of the 30 breast cancer patients included in the first group. For each patient, five TCPs were calculated: 1) of the dose distribution calculated with PBC‐MB (${\text{TCP}}_{\text{PBC}-\text{MB}}$), 2) of the dose distribution calculated with AAA (${\text{TCP}}_{\text{AAA}}$), 3) of the dose distribution calculated with Acuros‐M (${\text{TCP}}_{\text{Acuros}-M}$), 4) of the dose distribution calculated with Acuros‐W (${\text{TCP}}_{\text{Acuros}-W}$), and 5) of the dose distribution calculated with PBC‐Hom (${\text{TCP}}_{\text{PBC}-\text{Hom}}$). First, each homogeneous dose level of the dDVH was converted to a total dose delivered in 2 Gy fractions with the biological effective dose (BED) method,^(22)^ with α/β=4.6 Gy.^(23)^ Next, the TCP was calculated using the Poisson model for an inhomogeneous dose distribution^(24)^ using Eq. (1):

(1) $$\text{TCP}=(1/2)\sum_{i}v_{i}\exp(2\gamma_{50}(1-D_{i}/D_{50})/\ln2)$$

with $v_{i}$ the volume irradiated to a homogeneous dose $D_{i}$, $D_{50}$ the dose that locally controls 50% of the tumors, and $\gamma_{50}$ the expected change in TCP if the dose changes by 1% around $D_{50}$. In this study the parameters for adjuvant T1‐T2 WBRT were used: $D_{50}=30.89\;\text{Gy}$ and $\gamma_{50}=1.3\%/\%$ ^(25)^ For each dose calculation algorithm, the average TCP was calculated. The differences between the average TCPs were considered statistically significant if p≤0.05/4 (using the paired *t*‐test with Bonferroni correction for multiple testing).

For the same 30 patients, four ${\text{NTCPs}}_{\text{Fibrosis}}$ were calculated: 1) of the dose distribution calculated with PBC‐MB (${\text{NTCP}}_{\text{Fibrosis},\text{PBC}-\text{MB}}$), 2) of the dose distribution calculated with AAA (${\text{NTCP}}_{\text{Fibrosis},\text{AAA}}$), 3) of the dose distribution calculated with Acuros‐M (${\text{NTCP}}_{\text{Fibrosis},\text{Acuros}-M}$), and 4) of the dose distribution calculated with Acuros‐W (${\text{NTCP}}_{\text{Fibrosis},\text{Acuros}-W}$). Again, first the BED method^(22)^ was applied with α/β=3 Gy.^(26)^ Next, the NTCP was calculated using the relative seriality model for inhomogeneous dose distributions^(27)^ using Eq. (2):

(2) $$\text{NTCP}={\left[1-\prod {\left(1-{\left[2^{-e^{e\gamma (1-D_{i}/D_{50})}}\right]}^{S}\right)}^{v_{i}/V}\right]}^{1/s}$$

with $v_{i}$ the volume irradiated to a homogeneous dose $D_{i}$, V the total volume, $D_{50}$ the uniform dose that causes 50% probability of complication, $\gamma_{50}$ the slope of the curve, and s the relative seriality. ${\text{NTCP}}_{\text{Fibrosis}}$ five‐year post‐treatment was calculated with $D_{50}=62.4\;\text{Gy}$, $\gamma_{50}=1.47\%/\%$, and s=0.12.^(28)^ For each dose calculation algorithm, the average ${\text{NTCP}}_{\text{Fibrosis}}$ was calculated. The differences between the average ${\text{NTCPs}}_{\text{Fibrosis}}$ were considered statistically significant if p≤0.05/3 (using the paired *t*‐test with Bonferroni correction for multiple testing).

**Table 1:** Characteristics and tumor stage of the first group of breast cancer patients (N=30). Average difference ± standard deviation of the patient's age, volume of the ${\text{CTV}}_{\text{Breast}}$ ($V_{\text{Breast}}$), volume of the adipose tissue in the ${\text{CTV}}_{\text{Breast}}$ ($V_{\text{Adipose}}$), and volume of the muscle tissue in the ${\text{CTV}}_{\text{Breast}}$ ($V_{\text{Muscle}}$). $V_{\text{Adipose}}$ and $V_{\text{Muscle}}$ are determined using the mass density ranges $(0.590,0.985)g/{\text{cm}}_{3}$ ^(2)^ and $(0.985,1.075)g/{\text{cm}}_{3}$ ^(2)^, respectively

	*Patient Characteristics*			
*Age (yr)*	$V_{Breast}(cm^{3})$	$V_{Adipose}\;(cm^{3})$	$V_{Muscle}\;(cm^{3})$	*Stage*
55.5±10.3	508.4±251.5	470.9±251.4	36.2±31.5	#pTis=11
				#pT1=17
				#pT2=2

${\text{CTV}}_{\text{Breast}}=\text{clinical breast volume}$.

### E. Aim 2: Determination of algorithm‐specific TCP and ${\text{NTCP}}_{\text{Fibrosis}}$ parameters

As the TCP and the ${\text{NTCP}}_{\text{Fibrosis}}$ should be independent of the algorithm, significant differences were eliminated. Algorithm‐specific $D_{50}$ and $y_{50}$ parameters were determined with the least square fit — the difference between the known clinical outcome of the reference dose distribution (i.e., the dose distribution calculated for the clinical follow‐up study) and the clinical outcome of each non‐reference dose distribution (i.e., the dose distribution with unknown clinical outcome) was minimized. The α/β ratios and the seriality of the breast tissue were kept constant.

The PBC‐MB dose distribution has known ${\text{NTCP}}_{\text{Fibrosis}}$ outcome,^(28)^ and was considered as the reference dose distribution during least square fitting for ${\text{NTCP}}_{\text{Fibrosis}}$ parameter determination. In contrast, there is little knowledge on the reference dose distribution for TCP calculation, as the clinical follow‐up study was performed before 1976.^(29)^ In the 1960s and ‘70s, 3D treatment planning was very rare, and the first publications on the type‐a^(30)^ and the type‐b^(3)^ dose‐calculation algorithms date from 1992 and 1989, respectively. During the clinical follow‐up study, only the prescribed dose was known. Hence, the TCP of the homogeneous delivery of the prescription dose,^(24)^ (i.e., 93.4%) was the reference TCP for further investigation, starting with its comparison to the mean ${\text{TCP}}_{\text{PBC}-\text{Hom}}$, using the Student's *t*‐test.

### F. Aim 3: Validation of the algorithm‐specific parameters

The second group of 30 breast cancer patients was used to validate the algorithm‐specific TCP and ${\text{NTCP}}_{\text{Fibrosis}}$ parameters. For each patient the $(N){\text{TCP}}_{(\text{Fibrosis},)\text{PBC}-\text{MB}}$, $(N){\text{TCP}}_{(\text{Fibrosis},)\text{AAA}}$, $(N){\text{TCP}}_{(\text{Fibrosis},)\text{Acuros}-M}$, and $(N){\text{TCP}}_{(\text{Fibrosis},)\text{Acuros}-W}$ were calculated, as described above (see Aim 1), using the algorithm‐specific parameters derived in this study (see Aim 2).

## III. RESULTS

**Figure 2:**
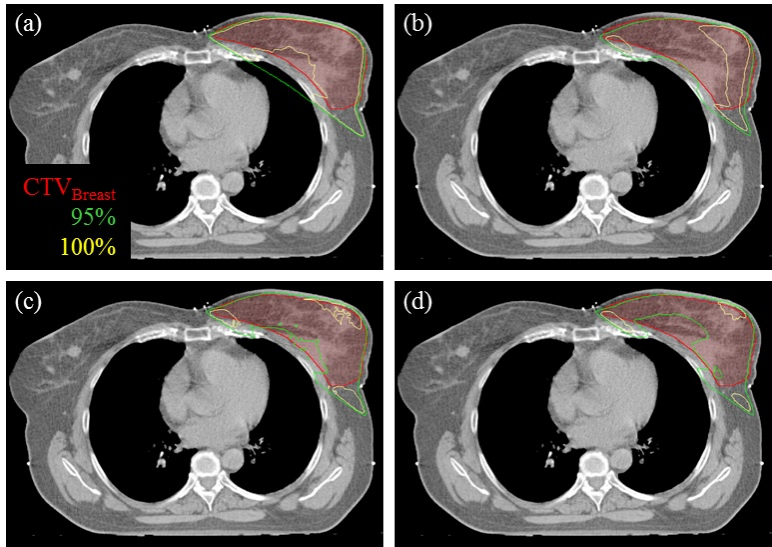
Transversal dose distributions in the breast: (a) PBC‐MB, (b) AAA, (c) Acuros‐M, (d) Acuros‐W. PBC−MB= pencil beam convolution algorithm with modified Batho heterogeneity correction; AAA=analytical anisotropic algorithm; Acuros−M=Acuros XB algorithm with dose‐to‐medium dose reporting; Acuros−W=Acuros XB algorithm with dose‐to‐water dose reporting; ${\text{CTV}}_{\text{Breast}}=\text{clinical target volume of the breast}$.

Figure 2 shows a transversal PBC‐MB, AAA, Acuros‐M, and Acuros‐W dose distribution in the breast. The largest differences between the dose distributions were found for the 95% and the 100% isodoses which shrink into the breast. The ordering of increasing shrinkage compared to the PBC‐MB dose distribution is: AAA, Acuros‐M, and Acuros‐W. The dose distributions were representative for the 3D dose distributions in each patient.

A summary of the differences between the cumulative dose‐volume histograms (cDVH) is given in Table 2.

**Table 2:** Average ± standard deviation for two dose distribution pairs in the ${\text{CTV}}_{\text{Breast}}$

	$\Delta D_{Mean}\;(Gy)$	$\Delta V_{90\%}\;(\%)$	$\Delta V_{95\%}\;(\%)$	$\Delta V_{100\%}\;(\%)$	$\Delta V_{105\%}\;(\%)$
AAA ‐ (PBC‐MB) (N=30)	‐0.9^a^±0.2	‐0.6^a^±0.5	‐5.9^a^±5.9	‐24.9^a^±7.8	‐4.4^a^±3.7
(Acuros‐M) ‐ AAA (N = 30)	‐0.3^a^±0.2	0.9^a^±1.0	‐1.1±5.0	‐11.0^a^±4.0	‐1.9^a^±2.3

a Statistically significant differences (p<0.05/2 with paired *t*‐test and Bonferroni correction for multiple testing). ${\text{CTV}}_{\text{Breast}}=\text{clinical breast volume}$; $D_{\text{mean}}=\text{mean dose}$; $V_{x\%}=\text{volume receiving}>x\%\;\text{of the prescription dose}$; PBC−MB= pencil beam convolution algorithm with modified Batho heterogeneity correction; AAA=analytical anisotropic algorithm; Acuros−M=Acuros XB algorithm with dose‐to‐medium dose reporting.

### A. Aim 1: Assessment of algorithm impact on TCP and ${\text{NTCP}}_{\text{Fibrosis}}$

The differences between the algorithms had a significant impact on the TCPs (Table 3): ${\text{TCP}}_{\text{PBC}-\text{MB}}$ was 1% higher than ${\text{TCP}}_{\text{AAA}}$ (p≪0.001), 1.3% higher than ${\text{TCP}}_{\text{Acuros}-M}$ (p≪0.001), and 2.1% higher than ${\text{TCP}}_{\text{Acuros}-W}$ (p≪0.001). Moreover, the differences between the algorithms resulted in significantly different ${\text{NTCPs}}_{\text{Fibrosis}}$ (Table 3): ${\text{NTCP}}_{\text{Fibrosis},\text{PBC}-\text{MB}}$ was 2.9% higher than ${\text{NTCP}}_{\text{Fibrosis},\text{AAA}}$ (p≪0.001) 4.1% higher than ${\text{NTCP}}_{\text{Fibrosis},\text{Acuros}-M}$ (p≪0.001), and 5.5% higher than ${\text{NTCP}}_{\text{Fibrosis},\text{Acuros}-W}$ (p≪0.001). The mutual differences between the ${\text{NTCPs}}_{\text{Fibrosis}}$ are 2.5 to 3 times larger than the mutual differences between the TCPs.

The differences between AAA and Acuros‐W were nonzero. The smallest TCP and ${\text{NTCP}}_{\text{Fibrosis}}$ differences were found between AAA and Acuros‐M.

**Table 3:** Average TCP and ${\text{NTCP}}_{\text{Fibrosis}}$ ± standard deviation, calculated with published model parameters. The table shows the results for the first group of 30 breast cancer patients. TCPs are calculated with $D_{50}=30.89\;\text{Gy}$,^(22)^ $\gamma_{50}=1.3\%/\%$,^(22)^ and α/β=4.6 Gy.^(20)^ ${\text{NTCPs}}_{\text{Fibrosis}}$ are calculated with $D_{50}=62.4\;\text{Gy}$,^(25)^ $\gamma_{50}=1.47\%/\%$,^(25)^ s=0.12,^(25)^ and α/β=3 Gy.(23)

	*PBC‐Hom* (N=30)	*PBC‐MB* (N=30)	*AAA* (N=30)	*Acuros‐M* (N=30)	*Acuros‐W* (N=30)
TCP (%)	93.3^a^±0.7	94.1±0.6	93.1^a^±0.7	92.8^a^±0.7	92.0^a^±0.9
NTCP_Fibrosis_ (%)	‐	21.6±2.3	18.7^a^±2.2	17.5^a^±2.0	16.1^a^±2.0

a Values are significantly different from PBC‐MB.

TCP=tumor control probability; ${\text{NTCP}}_{\text{Fibrosis}}=\text{normal tissue complication probability of moderate breast fibrosis}$; PBC−MB= pencil beam convolution algorithm with modified Batho heterogeneity correction; AAA=analytical anisotropic algorithm; Acuros−M=Acuros XB algorithm with dose‐to‐medium dose reporting; Acuros−W=Acuros XB algorithm with dose‐to‐water dose reporting.

### B. Aim 2: Determination of algorithm‐specific TCP and ${\text{NTCP}}_{\text{Fibrosis}}$ parameters

The average TCP of the PBC‐Hom dose distribution, 93.3% (±0.7%) (Table 3), was not significantly different from 93.4% (p=0.3) (i.e., the TCP of the homogeneous delivery of the prescription dose). This validates the assumption to consider the PBC‐Hom dose distribution as the reference dose distribution with known clinical outcome for T1‐T2 breast tumors, and to use it for the determination of the TCP parameters of the PBC‐MB, the AAA, the Acuros‐M, and the Acuros‐W algorithms (Table 4). α/β=4.6 Gy ^(23)^ was kept fixed.

PBC‐MB has known ${\text{NTCP}}_{\text{Fibrosis}}$ outcome.^(28)^ Least square fitting resulted in new parameters for the AAA, the Acuros‐M, and the Acuros‐W algorithms (Table 4). The relative seriality of the breast, s=0.12,^(28)^ and α/β=3 Gy ^(26)^ were kept fixed.

**Figure 3:**
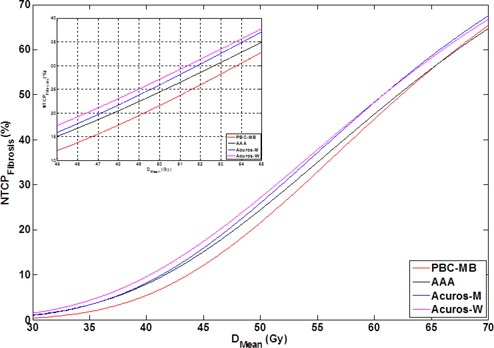
${\text{NTCP}}_{\text{Fibrosis}}$ as function of the mean dose in the ${\text{PTV}}_{\text{Breast}}$ for each dose calculation algorithm. The insert shows the ${\text{NTCP}}_{\text{Fibrosis}}$ curves in the clinically relevant dose range. ${\text{NTCP}}_{\text{Fibrosis}}=\text{normal tissue complication probability of moderate breast fibrosis}$; ${\text{PTV}}_{\text{Breast}}=\text{planning target volume of the breast}$; $D_{\text{Mean}}=\text{mean dose}$; PBC−MB= pencil beam convolution algorithm with modified Batho heterogeneity correction; AAA=analytical anisotropic algorithm; Acuros−M=Acuros XB algorithm with dose‐to‐medium dose reporting; Acuros−W=Acuros XB algorithm with dose‐to‐water dose reporting.

The $D_{50}$ for the TCP and the ${\text{NTCP}}_{\text{Fibrosis}}$ calculation decreased, compared to the value determined during the clinical follow‐up studies, meaning that both the probability of tumor control and of moderate breast fibrosis occur at a lower dose than first thought. ${\text{NTCP}}_{\text{Fibrosis}}$ least square fitting also altered the slope of the curve (i.e., $\gamma_{50}$, as is illustrated in Fig. 3). This is due to the limited clinical data in the low‐ and high‐dose regions.

**Table 4:** Algorithm‐specific model parameters for TCP and ${\text{NTCP}}_{\text{Fibrosis}}$ calculation

	$TCP:(D_{50},\gamma_{50},\alpha /\beta )$	$NTCP_{Fbrosis}:(D_{50},\gamma_{50},s,\alpha /\beta )$
PBC‐MB	(30.59 Gy, 1.2%/%, 4.6 Gy^(23)^)	(62.4 Gy,^(28)^ 1.47%/%,^(28)^ 0.12,^(28)^ 3 Gy^(26)^)
AAA	(29.82 Gy, 1.3%/%, 4.6 Gy^(23)^)	(62.1 Gy, 1.34%/%, 0.12,^(28)^ 3 Gy^(26)^)
Acuros‐M	(29.60 Gy, 1.2%/%, 4.6 Gy^(23)^)	(60.8 Gy, 1.38%/%, 0.12,^(28)^ 3 Gy^(26)^)
Acuros‐W	(29.91 Gy, 1.3%/%, 4.6 Gy^(23)^)	(60.8 Gy, 1.31%/%, 0.12,^(28)^ 3 Gy^(26)^)

TCP=tumor control probability; ${\text{NTCP}}_{\text{Fibrosis}}=\text{normal tissue complication probability of moderate breast fibrosis}$; PBC−MB= pencil beam convolution algorithm with modified Batho heterogeneity correction; AAA=analytical anisotropic algorithm; Acuros−M=Acuros XB algorithm with dose‐to‐medium dose reporting; Acuros−W=Acuros XB algorithm with dose‐to‐water dose reporting.

### C. Aim 3: Validation of the algorithm‐specific TCP and ${\text{NTCP}}_{\text{Fibrosis}}$ parameters

Application of the algorithm‐specific parameters to the second group of 30 breast cancer patients resulted in comparable predicted treatment outcomes, irrespective of the dose calculation algorithm (Table 5). This confirmed the elimination of the radiobiologically significant impact of the differences between the dose calculation algorithms and, hence, the appropriateness of the new TCP and ${\text{NTCP}}_{\text{Fibrosis}}$ parameters.

**Table 5:** Average TCP and ${\text{NTCP}}_{\text{Fibrosis}}$ ± standard deviation, calculated with algorithm‐specific model parameters. The table shows the results for the second group of 30 breast cancer patients. The TCPs and the ${\text{NTCPs}}_{\text{Fibrosis}}$ are calculated with the algorithm‐specific parameters from Table 4

	*TCP (%)*	$NTCP_{Fibrosis}(\%)$
PBC‐MB	92.8±0.6	20.1±2.6
AAA	92.8±0.6	20.1±2.0
Acuros‐M	92.8±0.6	20.4±2.0
Acuros‐W	92.7±1.1	20.3±2.4

TCP=tumor control probability; ${\text{NTCP}}_{\text{Fibrosis}}=\text{normal tissue complication probability of moderate breast fibrosis}$; PBC−MB= pencil beam convolution algorithm with modified Batho heterogeneity correction; AAA=analytical anisotropic algorithm; Acuros−M=Acuros XB algorithm with dose‐to‐medium dose reporting; Acuros−W=Acuros XB algorithm with dose‐to‐water dose reporting.

## IV. DISCUSSION

In this study, we clearly showed that the photon dose calculation algorithm used in whole breast radiotherapy has radiobiological and, therefore, clinical impact. This is the first study quantifying the radiobiological impact of the differences between the physical dose distributions in the breast. Our findings of the latter are in agreement with literature:^1^, ^7^, ^15^, ^16^, ^19^ the breast dose distribution calculated with the more advanced algorithms shrinks, compared to the dose distribution calculated with PBC‐MB.

Two recent studies have quantified the difference between the AAA and PBC‐MB dose distributions. Basran et al.^(14)^ and Yoo et al.^(16)^ found a significant decrease of the ${\text{CTV}}_{\text{Breast}}$ volume receiving at least 95% of the prescription dose, 10.5% and 5.9%±2.7%, respectively, when AAA is compared to PBC‐MB. Yoo and colleagues also reported significant decreases of the ${\text{CTV}}_{\text{Breast}}$ volume receiving at least 100% and 105% of the prescription dose, 14.9%±8.2% and 7.3%±5.0%, respectively. We found significant decreases of the ${\text{CTV}}_{\text{Breast}}$ volume receiving 95% and 105% of the prescription dose comparable to the study of Yoo et al.:^(16)^ 5.9%±5.9% and 4.4%±3.7%, respectively, (Table 5). In contrast, the decrease in ${\text{CTV}}_{\text{Breast}}$ volume receiving at least the prescription dose is 10% larger than that found by Yoo et al.: ^(16)^ 24.9%±7.8% (Table 5).

As the Acuros XB algorithm is recently implemented in Eclipse, while the use of PBC‐MB is almost erased by algorithms that take the lateral electron transport better into account, the comparison between PBC‐MB and Acuros XB is lacking. One study quantifies the difference between the AAA and the Acuros‐M dose distributions in the breast. Fogliata et al.^(15)^ reported, for both algorithms, a comparable mean ${\text{CTV}}_{\text{Breast}}$ dose and a comparable ${\text{CTV}}_{\text{Breast}}$ volume receiving at least 95% of the prescription dose. In contrast, the ${\text{CTV}}_{\text{Breast}}$ volume receiving at least 90% of the prescription dose increases with 2.7% if AAA is replaced by Acuros‐M.^(15)^ Basran et al.^(14)^ reported the difference between the AAA algorithm and Monte Carlo calculations, for which Acuros XB is a good alternative.^(9)^ Monte Carlo compared to AAA decreases the ${\text{CTV}}_{\text{Breast}}$ volume receiving 95% of the prescription dose with 1.3%.^(14)^ Our comparison of Acuros‐M with AAA revealed a significant increase, 0.9%±1%, and a decrease (1.1%±5%), although not significant, of the ${\text{CTV}}_{\text{Breast}}$ volume receiving at least 90% and 95% of the prescription dose, respectively (Table 2), if AAA is replaced by Acuros‐M. Hence, our findings are comparable to results described by Fogliata et al.^(15)^ and by Basran et al.^(14)^

We eliminated the radiobiological impact of the photon dose calculation algorithm by the determination of algorithm specific TCP and ${\text{NTCP}}_{\text{Fibrosis}}$ parameters using least square fitting. It is a less time‐consuming alternative to the clinical follow‐up of a large patient population. A limitation of the applied methodology is that the present treatment technique can never have a better tumor control or a higher complication rate than the clinical follow‐up study with which they are compared. Hence, even after eliminating the impact of the dose calculation algorithm, there is no exact knowledge of the breast tumor response to radiation, neither of the late cosmetic radiation effects — for example, moderate breast fibrosis. However, clinical follow‐up of our breast cancer patients showed a 10‐year relapse‐free survival rate of 96.9%.^(31)^ The difference with the TCP found by Ghossein et al.^(29)^ (i.e., 93.4%) can be ascribed to the boost of 16 Gy, which is included in our clinical follow‐up. Poortmans et al.^(32)^ reported that the boost decreases the 10‐year local recurrence rate with 4%. Clinical follow‐up on the moderate fibrosis of our treatment technique is lacking, but a value of 21.6%±2.3%, as predicted by Alexander et al.,^(28)^ seems acceptable. Jothy Basu et al.^(33)^ have reported a ${\text{NTCP}}_{\text{Fibrosis}}$ value of 25.66% for breast cancer patients treated with 25 fractions of 2 Gy with photon beams, followed by 8 boost fractions of 2 Gy with an electron beam. Although the algorithm‐specific parameters have clinical meaning, we still advise not to use the algorithm‐specific parameters for the prediction of the treatment outcome in an absolute sense, but only for the comparison of dose distributions — for example, to compare treatment plans or techniques or to quantify the radiobiological impact of off‐line and on‐line position correction protocols. An advantage of the TCP or NTCP comparison is that, in contrast to the cDVH analysis, only one parameter per region of interest is needed.

Except for the significant radiobiological impact of the advanced calculation algorithms compared to PBC‐MB, we also found a nonzero radiobiological impact of Acuros‐W compared to AAA. The smallest TCP and ${\text{NTCP}}_{\text{Fibrosis}}$ differences were found between Acuros‐M and AAA. This is due to the different methodologies used for volume scattering integration and heterogeneity modeling.^(2)^ In case of PBC‐MB or AAA dose calculation, Eclipse first converts the Hounsfield units of the CT scan to the electron density relative to water. During dose calculation, PBC‐MB only considers density corrections along the fan‐line (i.e., inhomogeneity corrections are based on an equivalent path length scaling). In contrast, dose calculation with AAA approximates the lateral electron transport and tissue heterogeneities are accounted for in an anisotropical way. In case of Acuros XB dose calculation, Eclipse first converts the Hounsfield units to mass density, such that the tissue‐specific elemental composition of the human body is taken into account during radiation transport calculation. Next, the dose is calculated according to the dose reporting mode. In case of dose to medium, the tissue composition is taken into account. In case of dose to water, the patient is considered as consisting of water. The water/medium stopping‐power ratio relates both dose reporting modes.^(34)^ The breast is mainly composed of adipose tissue (Table 1), which has a stopping‐power ratio of 0.978.^(35)^ This explains why Acuros‐W predicts the lowest breast dose and, hence, the lowest TCP and ${\text{NTCP}}_{\text{Fibrosis}}$.

This study shows that preclinical evaluation of advanced photon dose calculation algorithms in WBRT is important. Following the ICRU guidelines,^(36)^ the dose delivered to the target volumes should be between 95% and 107% of the prescription dose. We showed that the shrinkage of the 95% isodose of the more advanced dose calculation algorithms, compared to PBC‐MB, has significant clinical impact. Clinical experience is based on the PBC‐MB dose distributions and, more recently, on the AAA dose distributions. The first studies on the validation and evaluation of Acuros XB in homogeneous and heterogeneous phantoms have shown good agreement with measurements and Monte Carlo calculations.^9^, ^21^, ^37^ Acuros XB with dose‐to‐medium dose reporting has been shown to be superior to dose‐to‐water dose reporting, and to be an accurate alternative for Monte Carlo calculations.^(9)^ In agreement with the study of Ma and Li,^(38)^ we suggest dose to medium as the preferred dose reporting mode in WBRT for consistency with clinical practice. This opens the discussion on dose prescription in whole breast radiotherapy: Should the prescription dose be adjusted to the actually delivered dose, most accurately predicted by Acuros XB with dose‐to‐medium dose reporting? or, Should the actually delivered dose distribution be optimized to obtain the ICRU guidelines? The latter needs an increase in the number of MUs to compensate for the loss of scatter. However, this will also increase the probability of moderate breast fibrosis, which we showed to occur at a lower dose than first thought.

### V. CONCLUSIONS

Ignoring the lateral electron transport and the tissue composition in whole breast radiotherapy has radiobiological and, therefore, clinical impact. Using a large breast population and comparing several types of photon dose calculation algorithms, this work shows that the determination of algorithm‐specific TCP and ${\text{NTCP}}_{\text{Fibrosis}}$ parameters eliminates the radiobiological impact of the calculation algorithm.

## ACKNOWLEDGMENTS

The authors would like to thank the Myny‐Vanderpoorten Foundation for financial support.
